# Advanced therapy medicinal products: current and future perspectives

**DOI:** 10.3402/jmahp.v4.31036

**Published:** 2016-04-25

**Authors:** Eve Hanna, Cécile Rémuzat, Pascal Auquier, Mondher Toumi

**Affiliations:** 1 Public Health Department - Research Unit, EA 3279, Aix Marseille University, Marseille, France; 2 Creativ-Ceutical, Paris, France

**Keywords:** advanced therapy medicinal products, ATMPs, clinical trials, market access

## Abstract

**Background:**

Advanced therapy medicinal products (ATMPs) are innovative therapies that encompass gene therapy, somatic cell therapy, and tissue-engineered products. These therapies are expected to bring important health benefits, but also to substantially impact the pharmaceuticals budget.

**Objective:**

The aim of this study was to characterise the ATMPs in development and discuss future implications in terms of market access.

**Methods:**

Clinical trials were searched in the following databases: EudraCT (EU Drug Regulating Authorities Clinical Trials), ClinicalTrials.gov, and ICTRP (International Clinical Trials Registry Platform of the World Health Organization). Trials were classified by category of ATMP as defined by European regulation EC No. 1394/2007, as well as by development phase and disease area.

**Results:**

The database search identified 939 clinical trials investigating ATMPs (85% ongoing, 15% completed). The majority of trials were in the early stages (Phase I, I/II: 64.3%, Phase II, II/III: 27.9%, Phase 3: 6.9%). Per category of ATMP, we identified 53.6% of trials for somatic cell therapies, 22.8% for tissue-engineered products, 22.4% for gene therapies, and 1.2% for combined products (incorporating a medical device). Disease areas included cancer (24.8%), cardiovascular diseases (19.4%), musculoskeletal (10.5%), immune system and inflammation (11.5%), neurology (9.1%), and others. Of the trials, 47.2% enrolled fewer than 25 patients. Due to the complexity and specificity of ATMPs, new clinical trial methodologies are being considered (e.g., small sample size, non-randomised trials, single-arm trials, surrogate endpoints, integrated protocols, and adaptive designs). Evidence generation post-launch will become unavoidable to address payers’ expectations.

**Conclusion:**

ATMPs represent a fast-growing field of interest. Although most of the products are in an early development phase, the combined trial phase and the potential to cure severe chronic conditions suggest that ATMPs may reach the market earlier than standard therapies. Targeted therapies have opened the way for new trial methodologies, from which ATMPs could benefit to get early access. ATMPs may be the next source of major impact on payers’ drug budgets.

Advanced therapy medicinal products (ATMPs) constitute an innovative class of heterogeneous research driven biopharmaceuticals. This class encompasses gene therapy medicinal products (GTMPs), somatic cell therapy medicinal products (sCTMPs), tissue-engineered products (TEPs), and combined products (tissue or cell associated to a device) (1). The legal and regulatory framework for ATMPs in the European Union (EU) was established by the EU Commission in 2007 (Regulation EC No. 1394/2007) and first applied in December 2008 (2). The Committee for Advanced Therapies (CAT) of the European Medicines Agency plays an important role in the regulatory oversight of these products: among other responsibilities, it is the main scientific committee in charge of evaluating marketing authorisation (MA) applications for ATMPs and provides scientific recommendations for the classification of ATMPs (2, 3).

As defined by the regulation, GTMPs are products of biological origin containing recombinant nucleic acid(s) and that have a therapeutic, prophylactic, or diagnostic effect related directly to the recombinant nucleic acid sequence. sCTMPs are biological products that contain or consist of cells or tissues that have been subject to substantial manipulation or that are not intended to be used for the same essential function(s) in the recipient and the donor; the recipient and the donor could be the same person. TEPs contain or consist of engineered cells or tissues and are presented as having properties for, or are used in or administered to, human beings with a view to regenerating, repairing, or replacing human tissue (1, 4).

Eight years after the adoption of the regulation, only five ATMPs have been granted MA in the EU as of October 2015: one cell therapy, Sipuleucel-T (Provenge^®^, 2013) for metastatic castrate-resistant prostate cancer (5); one gene therapy, alipogene tiparvovec (Glybera^®^, 2012) for lipoprotein lipase deficiency (6); and three TEPs – autologous cartilage cells expanded *ex vivo* expressing specific marker proteins (Chondrocelect^®^, 2009) (7), matrix applied characterised autologous cultured chondrocytes (MACI^®^, 2013) for cartilage defects (8), and *ex vivo* expanded autologous human corneal epithelial cells containing stem cells (Holoclar^®^, 2015) for severe limbal stem-cell deficiency caused by burns to the eyes (9). However, the MA for MACI^®^ was suspended due to the closure of the EU manufacturing site (10), and the MA for Provenge^®^ was withdrawn due to the bankruptcy of the MA holder Dendreon (11, 12).

ATMPs hold great potential for reshaping the progression or the disability associated with multiple diseases such as Alzheimer's disease, Parkinson's disease, cancer, muscular dystrophy, and so on (13, 14), including an option for curing or reversing diseases known as untreatable today or that are just subject to symptomatic treatments. Maciulaitis et al. (15) identified 318 clinical trials for ATMPs registered in the EU Drug Regulating Authorities Clinical Trials (EudraCT) database between 2004 and 2010. In addition to Maciulaitis et al. (15), three studies focused on a specific ATMP segment at the global or UK level. The *Journal of Gene Medicine* gives access, through its Gene Therapy Clinical Trials Worldwide database, to the annual number of approved, ongoing, or completed gene therapy clinical trials worldwide (16). Trouson and Mc Donald (17) showed the progress in developing stem cells therapies and the challenges facing them. Bisson et al. (18) identified 41 ongoing cell therapy clinical trials in April 2014 in the United Kingdom; the majority were in an early phase and led by academics.

However, the development of ATMPs is a dynamic and fast-growing field; it has progressed greatly since the study by Maciulaitis et al. (15) identifying ATMP studies in 2010. The 2010 cut-off date of Maciulaitis et al. is likely outdated, and a more up-to-date study would be valuable for the scientific society, policy decision makers, and payers. Such a study would help payers to gain the awareness to anticipate the hypothetical short- and medium-term budget impact of such products.

If these products meet expectations and provide evidence of efficacy and effectiveness in the ongoing clinical trials, we assume that a large number of ATMPs in development may reach the market. Given the high additional value they may offer to patients and society, as well as the high prices anticipated for these products, they may have a substantial budget impact and pose a challenge for the sustainability of public health insurance in Europe.

The aim of this study was to identify the number of ATMPs in development, the sponsor type, the nature of the products, the targeted diseases, and the development stage and to provide objective information that may enlighten discussions and research for ATMPs including future implications in terms of market access and the potential impact on health insurance budgets. Such information would facilitate further reflection on new pricing and reimbursement policies to secure the sustainability of national health insurance in Europe.

## Materials and methods

### Data collection

Two independent researchers retrieved all clinical trials of ATMPs conducted between 1999 and June 2015 using three clinical trials databases: ClinicalTrials.gov, the International Clinical Trials Registry Platform (ICTRP) of the World Health Organization (WHO), and EudraCT. The same combinations of keywords were used for the three databases searches:

- For cell therapies: cell therapy, stem cell, cord blood, umbilical cord, bone marrow, cancer vaccine, tissue engineering, engineered cell, tissue engineered, mesenchymal cell, somatic cell, allogeneic cell, viable cell
- For gene therapies: gene therapy, recombinant nucleic acid, DNA therapy, cDNA, recombinant DNA, nucleic acid therapy, gene transfer, virus delivery, cancer immunotherapy, RNA therapy, tumour vaccine, genetic therapy, plasmid DNA, oligonucleotides, genetically modified microorganisms, genetically modified organisms, genetically modified cells

### Data extraction and selection

We designed specific data extraction forms using Microsoft Excel 2010 to extract the following clinical trials data: registration number, date of registration, title, status, phase, study design, target enrolment number, sponsor, disease, and last update date.

Duplicate studies with the same registration number were removed as well as all pre-clinical studies, Phase 0 (exploratory) studies, pilot studies, and observational studies.

We excluded trials that were not for ATMPs, and we classified the remaining trials by ATMP class, based on the definition of *ATMP* provided by the European regulation EC No. 1394/2007 (2):

GTMPs should fulfil the three following criteria:

- Have biological origin.
- Contain recombinant nucleic acid(s).
- The therapeutic, prophylactic, or diagnostic effect should relate directly to the recombinant nucleic acid sequence it contains or to the product of genetic expression of this sequence.

sCTMPs and TEPs both contain or consist of engineered cells or tissues. To be considered engineered, cells or tissues should fulfil at least one of the following criteria:

- Substantial manipulation: biological characteristics, physiological functions, or structural properties relevant for the intended regeneration, repair, or replacement are achieved during their manipulation.
- Non-homologous use: the cells and tissues are not intended to be used for the same essential function (s) in the recipient and the donor.

sCTMPs are presented as having properties for being used in or administered to human beings with a view to treating, preventing, or diagnosing a disease through the pharmacological, immunological, or metabolic action of its cells or tissues. TEPs are presented as having properties for, or being used in or administered to, human beings with a view to regenerating, repairing, or replacing human tissue (1, 19).

The classification was performed by two reviewers and discrepancies re-analysed. Any persistent discrepancy was resolved by consensus or, failing that, by arbitration with the support of a senior researcher skilled in pharmaceutical sciences and biologics.

### Data analysis

The data were sorted out by the following:

1. Sponsor status: commercial, non-commercial. For non-commercial sponsors, the corresponding clinical trials were classified into five settings: hospital, university, institute, medical centre, and government.
2. Development phase: Phase I, I/II, II, II/III, and III.
3. Pathology:
  - Cancer
  - Cardiovascular
  - Musculoskeletal
  - Immune system/inflammation
  - Neurology
  - Gastrointestinal diseases (GI)/diabetes
  - Ophthalmology
  - Pulmonology
  - Dermatology: wounds, ulcers
  - Haematology: anaemia, haemophilia
  - Others
4. Date range: the following date ranges were considered to assess the evolution over time of the number of clinical trials with ATMPs: 1999–2003; 2004–2010; 2011–2015.
5. The last update date was recorded for each trial.
6. Target enrolment number was classified according to the following range: <25, 25–50, 51–100, >100.

## Results

### Search results

**Fig. 1:**
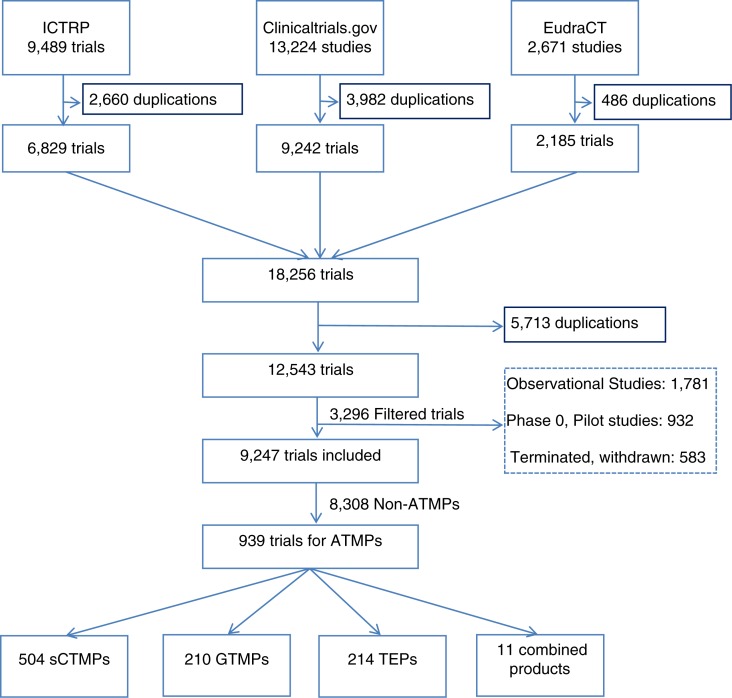
Flow chart of clinical trials identification and inclusion. This flow chart summarises the results obtained in each step of the study, starting by the database searches, the results after removing duplications from each database, then removing duplications from the combination of the results, including the filtered studies and the studies. ATMPs: advanced therapy medicinal products; sCTMPs: somatic cell therapy products; TEPs: tissue-engineered products; GTMPs: gene therapy medicinal products; ICTRP: International Clinical Trials Registry Platform.

The search strategy resulted in a total of 25,384 trials. After removing duplicates, observational studies, Phase 0 studies, and pilot studies, as well as terminated or withdrawn studies, 9,247 trials were considered for ATMP classification. Finally, we identified 939 clinical trials investigating ATMPs (Fig. 1).

### ATMP class

**Fig. 2:**
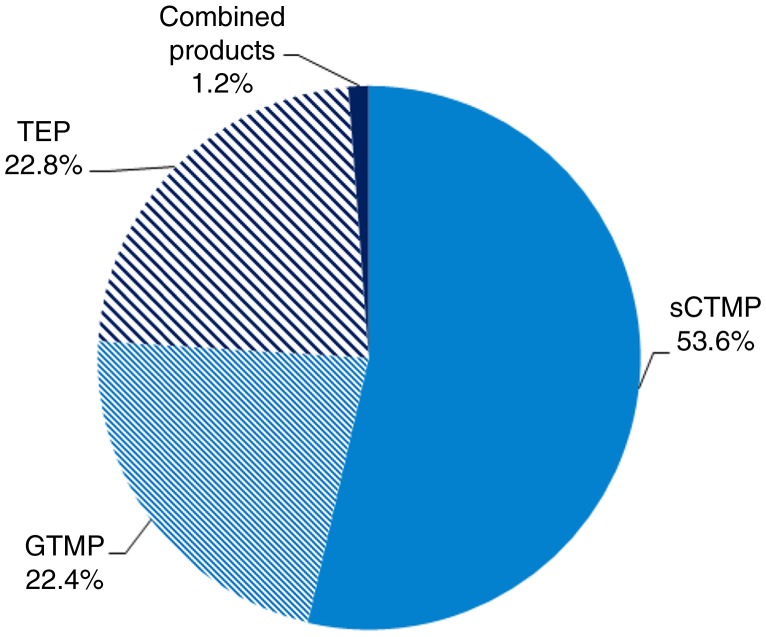
ATMP classification: percentages of gene therapy medicinal products (GTMPs), somatic cell therapy medicinal products (sCTMPs), tissue-engineered products (TEPs), and combined products.

Almost half of the medicinal products in development were somatic cell therapies (53.6%); the remainder were either TEPs (22.8%), gene therapies (22.4%), or combined products (representing only 1.2% of the products) (Fig. 2).

### Registration date and status

**Fig. 3:**
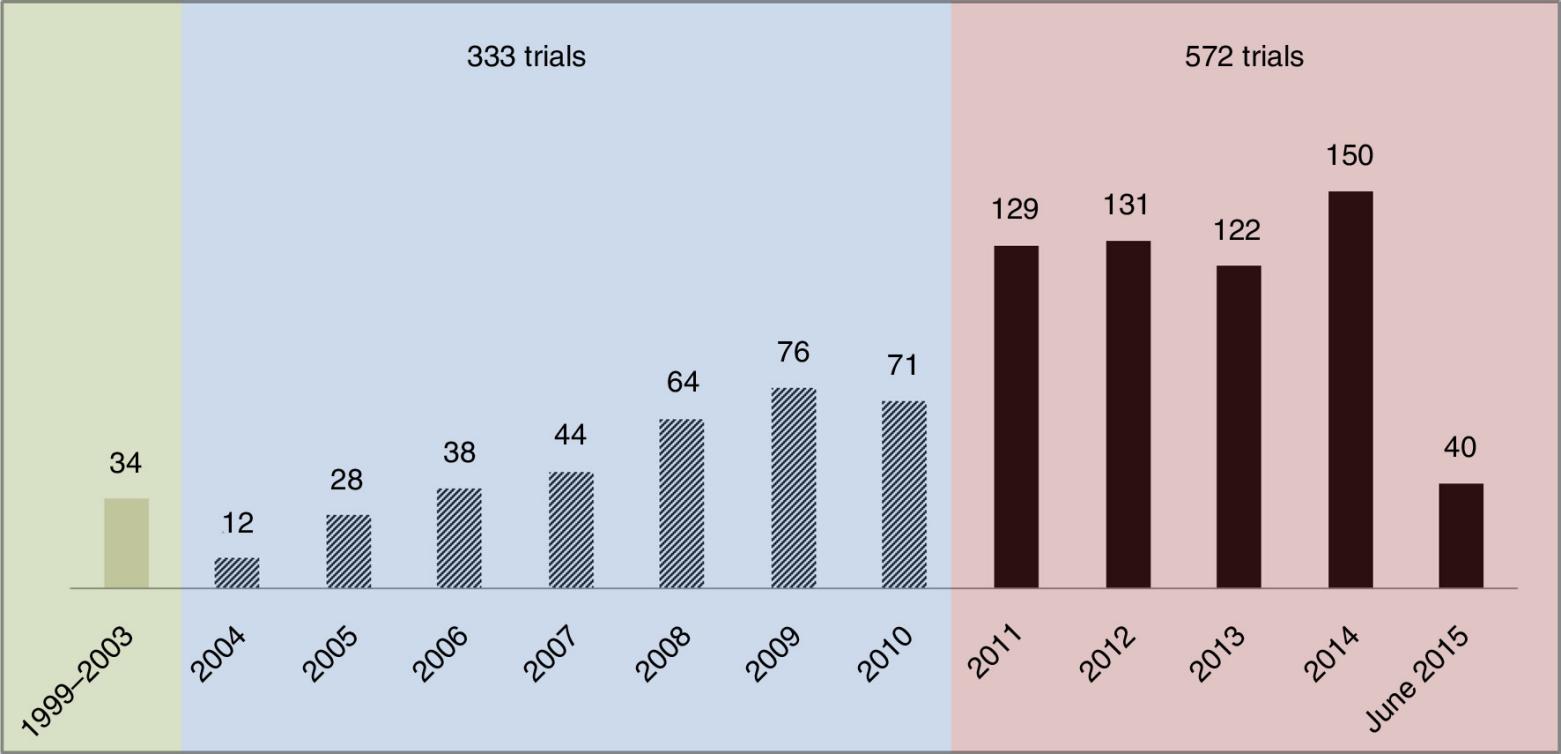
Number of ATMP trials registered by year between 2004 and June 2015 and by range: 1999–2003, 2004–2010, and 2011–2015.

**Fig. 4:**
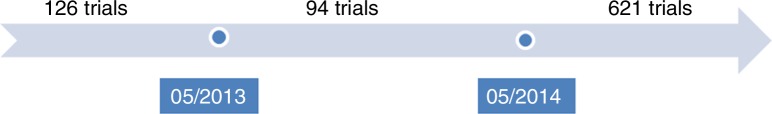
Number of ATMP trials updated recently (>05/2014), the trials updated between 05/2013 and 05/2014, and the trials having an update date of 2 years or older (<05/2013). *Note*: The update date was not reported in 98 trials, most of which were recently initiated studies. This figure shows the number of trials updated recently (>05/2014), the trials updated between 05/2013 and 05/2014 and the trials having an update date of 2 years or older (<05/2013).

Overall, the results showed that the number of ATMPs clinical trials has been consistently growing over the past 15 years. Of the 939 ATMP trials, 34 trials were registered in 1999–2003, compared to 333 in 2004–2010 and as many as 572 trials in 2011–2015 (Fig. 3). Of the trials, 85% were still ongoing and 15% were completed. Two-thirds of the ATMP trials (621 trials) included had a recent last update date, suggesting that they were still active, and 126 (13.4%) had an update date that was 2 years or more in the past (Fig. 4). Four hundred forty-four trials (47.2%) enrolled fewer than 25 patients.

### Targeted disease

Although these therapies are being developed to target several different diseases, oncology remained the dominant therapeutic area, accounting for 24.8% of the trials identified: leukaemia/lymphoma/myeloma (30.9%), skin cancer (10.3%), prostate cancer (9.9%), brain cancer (8.2%), cancer of the GI system (7.7%), bladder and renal cancer (6.8%), nasopharyngeal and lung cancer (4.7%), breast cancer (3.9%), and others (6.0%). The type of cancer was not specified in 11.6% of the oncology trials. Cardiovascular diseases represented the second biggest therapeutic area, making up 19.4% of the trials: heart failure (ischemic and non-ischemic)/cardiomyopathy (31.3%), limb ischemia and peripheral arterial disease (24.2%), myocardial infarction/coronary artery diseases (23.6%), stroke (11.5%), and others (9.3%). Many other disease areas were identified: inflammation (11.5%); musculoskeletal system diseases (10.5%); neurology (9.1%); GI disease, diabetes (5.2% all together); ophthalmology (4.7%); pulmonology (3.4%); dermatology (3.1%); haematology (2.1%); and other therapeutic areas (6.2%) (Tables 1 and 2).

**Table 1:** Classification of ATMPs trials by disease area and phase of development

	Phase I and I/II	Phase II and II/III	Phase III	NA	Total
Cancer	146 (24.2%)	69 (26.4%)	18 (27.8%)		233 (24.8%)
Cardiovascular diseases	104 (17.2%)	67 (25.7%)	11 (16.7%)		182 (19.4%)
Immune system/inflammation	68 (11.3%)	29 (11.1%)	9 (13.9%)	2 (22.2%)	108 (11.5%)
Musculoskeletal system	59 (9.8%)	25 (9.6%)	9 (13.9%)	6 (66.7%)	99 (10.5%)
Neurology	61 (10.1%)	23 (8.8%)	1 (1.5%)		85 (9.1%)
GI diseases and diabetes	25 (4.1%)	15 (5.7%)	8 (12.3%)	1 (11.1%)	49 (5.2%)
Ophthalmology	34 (5.6%)	7 (2.7%)	3 (4.6%)		44 (4.7%)
Pulmonology	25 (4.1%)	6 (2.3%)	1 (1.5%)		32 (3.4%)
Dermatology	19 (3.1%)	7 (2.7%)	3 (4.6%)		29 (3.1%)
Haematology	16 (2.6%)	4 (1.5%)	0		20 (2.1%)
Others	47 (7.8%)	9 (3.4%)	2 (3.1%)		58 (6.2%)
Total	604 (64.3%)	261 (27.9%)	65 (6.9%)	9 (0.9%)	939 (100%)

GI diseases: gastrointestinal diseases.

*Notes:* The table shows the number of ATMP trials in each disease area: cancer, cardiovascular diseases, immune system/inflammation, musculoskeletal system, neurology, GI diseases and diabetes, ophthalmology, pulmonology, dermatology, haematology, and others. In addition, it shows the percentage of ATMPs targeting every indication by phase of development.

**Table 2:** Percentage of trials in each disease area

Disease area	Diseases	Number of trials
Cancer	Leukaemia/lymphoma/myeloma	72 (30.9%)
	Skin cancer	24 (10.3%)
	Prostate cancer	23 (9.9%)
	Brain cancer	19 (8.2%)
	Gastrointestinal system cancer	18 (7.7%)
	Bladder or renal cancer	16 (6.8%)
	Respiratory system (nasopharyngeal, lung) cancer	11 (4.7%)
	Breast cancer	9 (3.9%)
	Other	14 (6.0%)
	Blank (type of cancer not specified)	27 (11.6%)
Total		233
Cardiovascular diseases	Heart failure, ischemic and non-ischemic/cardiomyopathy	57 (31.3%)
	Limb ischemia and peripheral arterial disease	44 (24.2%)
	Myocardial infarction/coronary artery diseases	43 (23.6%)
	Stroke	21 (11.5%)
	Other	17 (9.3%)
Total		182
Musculoskeletal	Bone defects	46 (46.5%)
diseases	Muscular dystrophy	28 (28.3%)
	Cartilage defects	22 (22.2%)
	Tendinopathy/ligament defects	3 (3.0%)
Total		99
Inflammation/	Diverse inflammations	39 (36.1%)
immune	Arthritis/spondylitis	29 (26.8%)
system	Crohn's disease	23 (21.4%)
	Lupus	4 (3.7%)
	Other	13 (12.0%)
Total		108
Other	XCGD	34 (58.6%)
	Enzyme deficiency/lysosome	20 (34.6%)
	Infertility	1 (1.7%)
	Vocal cord	1 (1.7%)
	Ear membrane	2 (3.4%)
Total		58

XCGD: X-linked chronic granulomatous disease.

*Notes:* This table presents the percentage of each disease included in the therapeutic areas: cancer, cardiovascular diseases, musculoskeletal diseases, inflammation/immune system, and others.

The majority of identified ATMP trials were in the early stages of development, as shown in Table 1, with 64.3% of the trials in Phase I or I/II, 27.9% in Phase II or II/III, and 6.9% (65 trials) in Phase III.

One-quarter of Phase I and I/II trials targeted cancer, 17.2% cardiovascular diseases, and around 10% were in the areas of immunology and inflammation, musculoskeletal diseases, and neurology.

Similarly, cancer and cardiovascular diseases were targeted by around 25% of Phase II and II/III trials each. Of Phase III trials, 27.8% were ATMPs targeting cancers and 16.7% cardiovascular diseases (Table 1).

### Sponsor status

**Fig. 5:**
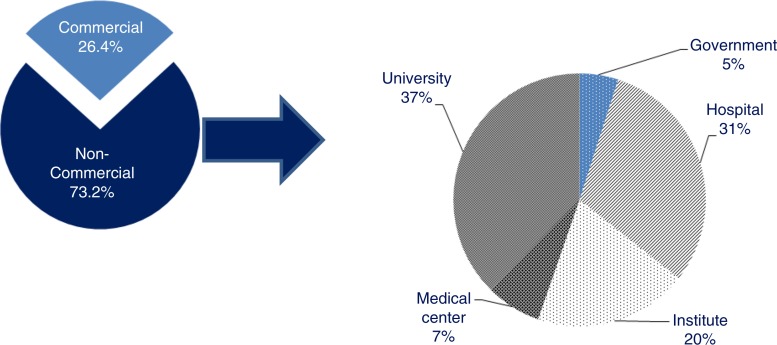
Distribution of the sponsors of ATMP clinical trials. The first pie chart shows the distribution of the sponsors of ATMPs trials between commercial and non-commercial sponsors; the second pie chart shows the distribution of non-commercial sponsors between: university, medical centres, institutes, hospitals, and the government. *Note*: Sponsor was not specified in 0.4% of the trials.

The vast majority of the trials were sponsored by non-commercial sponsors (73.2%). Universities (37%), hospitals (31%), and public or para-public research institutes (20%) were the main non-commercial sponsors, whereas government and medical centres represented respectively 5 and 7% of non-commercial sponsors (Fig. 5). Interestingly, when comparing the sponsor status and the development phase, results showed that 20.5% of trials in Phase I or I/II were sponsored by commercial sponsors, whereas this percentage rose to 53.8% of Phase III trials (Table 3).

**Table 3:** Classification of trials by sponsor status and phase of development

	Phase I and I/II	Phase II and II/III	Phase III	Phase not specified	Total
Commercial	124 (20.5%)	85 (32.6%)	35 (53.8%)	4 (44.4%)	248 (26.4%)
Non-commercial	480 (79.5%)	174 (66.6%)	30 (46.2%)	3 (33.4%)	687 (73.2%)
Sponsor not specified		2 (0.8%)		2 (22.2%)	4 (0.4%)
Total	604 (64.3%)	261 (27.9%)	65 (6.9%)	9 (0.9%)	939 (100%)

*Notes:* The table shows the number of trials sponsored by a commercial and non-commercial sponsor and the sponsor status in each phase of development.

## Discussion

ATMPs are a new class of biopharmaceuticals. Eight years after the adoption of the regulation, only five products have obtained regulatory approval in the EU and achieved limited success in securing reimbursement (20). This fact suggests a low impact of ATMPs to date on health insurance budgets but also on patients’ health. To identify the number and characteristics of ATMPs in development, we searched three different clinical trial databases and extracted the trials using specific keywords. We believe we have likely captured most of the ATMPs in development. We used the following intervals of time: 1999–2003, which was the period before the initiation of EudraCT; 2004–2010, which was the interval of time between the initiation of EudraCT and the end of the first term of the CAT, considered a milestone; and the adoption of the CAT work programme (2010–2015) that aims to foster development of ATMPs and bring more ATMPs to the market. Comparison of the time periods 2004–2010 and 2011–2015 can help to reflect the impact of the regulation on the development of ATMPs.

Our results showed that 939 ATMP clinical trials were being conducted in different disease areas. Cancer was the first indication targeted by ATMPs; almost a quarter of the trials were for ATMPs developed to treat cancers, and 19.4% of the trials were for cardiovascular diseases. The majority of these trials were ongoing (85%) and in the early stages of development (92.2%: Phase I, combined I/II, II, combined II/III), and 65 trials (6.9%) were in Phase III, suggesting they had a successful Phase II with substantial chances to reach the market in the coming 5 years. However, to our knowledge since it is a new therapeutic class, there is no success rate per development phase for ATMPs that would allow an estimate of the potential ones likely to reach the market. Two-thirds of the trials had a recent update date, suggesting that they were still active. These results support our hypothesis that a fast-growing number of ATMPs may ultimately impact the national health insurance budget in Europe. Moreover, 932 studies are currently in a very early phase, such as pilot studies, and were not considered for this research. These studies will continue to fuel the number of ATMPs in development in the coming years.

Our results are consistent with the results of Maciulaitis et al. (15), who identified 318 clinical trials for ATMPs between 2004 and 2010; during this same time period, we identified 333 clinical trials. This 5% increase may be based on the use of multiple databases, or just on differences in ATMP classification. In fact, the study by Maciulaitis et al. (15) was based solely on the EudraCT database; therefore, it reflected the situation only in Europe. In contrast, we used two international databases (ICTRP and ClinicalTrials.gov) in addition to EudraCT.

Consistent with Maciulaitis et al., our findings show that the vast majority of ATMP sponsors were non-commercial (73.2%). In addition, we showed that 53.8% of Phase III trials were sponsored by commercial sponsors, whereas only 20% of Phase I and I/II trials had commercial sponsors. In fact, non-commercial sponsors may have a limited budget and limited experience to achieve the development of their products. Products initially developed by non-commercial sponsors may either be moved to a spin-off organisation or be licensed to a pharmaceutical industry to achieve their development in later phases and obtain MA. This could explain the gap in development status between commercial and non-commercial organisations. This assumption was not tested.

Our study showed that the number of ATMPs in development has increased considerably, from 12 trials in 2004 to 150 in 2014 (Fig. 3). Developing ATMPs is a complex process because of technical obstacles and uncertainties. Due to the complexity and specificity of ATMPs, new clinical trial methodologies are expected to be considered, similarly to what is discussed for oncology products and orphan drugs (e.g., small sample size, non-randomised trials, single-arm trials, surrogate end points, integrated protocols, combined Phase II/III, and adaptive designs) (21). Therefore, at the time of launch, payers may end up with insufficient information to assess the potential value of the products being launched. Evidence generation post-launch will likely become unavoidable to address payers’ uncertainties. Manufacturers developing such products should bear in mind the need to inform payers early on about their product value. They should be prepared to collect long-term follow-up information and consider post-launch studies and eventually coverage with evidence development with or without escrow agreements.

Despite their potential for improving efficacy, ATMPs may encounter substantial hurdles to reach the market if the manufacturer has not appropriately prepared the market access strategy and launch sequence. Currently, the increasing number of highly effective therapies approved in the EU (22) is creating increasing financial pressure on healthcare budgets during a period of recovery after a financial crisis and flattening of gross domestic product growth (23). The payers are facing the challenge of creating a balance between ensuring the financial sustainability of the healthcare system and encouraging the innovation and development of new therapies to address unmet needs. This situation, together with poor preparation of pharmaceutical companies, may explain the limited success of the five approved ATMPs to secure reimbursement in the EU (20). The manufacturer of alipogene tiparvovec (Glybera^®^), the first gene therapy, is seeking a price of 53,000 euros/vial, thus 1.1 million euros per patient. Although it targets a small population group, it will create a substantial financial impact by adding it to the numerous orphan drugs reaching the market at a high price (24, 25). Glybera^®^ is not reimbursed in the EU; after its assessment, the Federal Joint Committee, Der Gemeinsame Bundesausschuss (G-BA) could not make a final conclusion on its benefits due to the limited data submitted by the manufacturer (26). Sipuleucel-T (Provenge^®^), an ATMP used for metastatic castrate-resistant prostate cancer, was priced at $90,000 for three doses in the United States (27). Because of the high price requested by the manufacturer, the evidence provided by the manufacturer to health technology assessment agencies was subject to very high scrutiny. It was denied reimbursement in Europe; the National Institute for Health and Care Excellence concluded that Provenge^®^ did not demonstrate either additional benefit or cost-effectiveness compared to the best supportive care, and G-BA concluded there was a ‘non-quantifiable’ added benefit (28, 29). Ultimately, Dendreon, the company manufacturing Provenge^®^, went bankrupt, primarily (but not only) because of the poor pricing and market access strategy (12). Due to widespread incidence and unmet medical need, oncology and cardiology constitute the target for a significant proportion of ATMPs in clinical development. As we have shown, these two therapeutic areas cover almost half of the ATMPs in development (24.8 and 19.4%, respectively). Around 30% of Phase III trials are for cancer; this shows that oncology advanced therapies are the closest to fuel ATMPs market growth. In recent years, cancer drug prices have been skyrocketing, placing huge funding dilemmas on healthcare systems (30). Cancer therapies cost the EU 124 billion euros each year (31). If ATMPs in development meet expectations, the manufacturers will be targeting premium prices, which will create a dramatic impact on payers’ budgets (32). EU5 payers are reluctant to pay premium prices with immature data, while the benefits they are paying for are expected to materialise beyond the duration of clinical trials (33).

Resource utilisation prioritisation will increasingly be required for the introduction of those new medicines and should be transparent and driven by society preferences (32).

The recent example of sofosbuvir showed how unprepared health authorities are and how inappropriate the payers’ decision-making criteria are, when it is necessary to make a decision on a high-value product with a major budget impact. Payers tend to deviate from their own established decision-making rules, operating through exceptional rules and capping the drug class expenditure without considering the overall disease expenditure (34).

This situation is likely to be replicated many dozens of times in the coming decade. We foresee multiple ATMPs reaching the market with limited clinical evidence but a potential for very high benefit, making it extremely difficult for payers to deny access. Society will exert heavy pressure on the politicians in charge of the administration and on policy makers to get access to those products.

Pricing regulations need to be reconsidered in the light of that situation in order to take into account the growing pipeline of innovative high-value products approaching the market in the coming decade.

There are some limitations of our study. First, the trials initiated before the implementation of the regulation may not be registered in the databases. However, very few products that would qualify as ATMPs were in development at that time, limiting the potential for this fact to alter the outcome of this research. Second, selection bias is possible, actually in ClinicalTrials.gov and ICTRP, as the investigational products were not classified and we did the classification following the definition in the regulation. The double-blind classification of ATMPs should have prevented significant misclassification.

## Conclusion

ATMPs represent a fast-growing field of interest. The large number of ATMPs in development is likely to continue to grow, fuelled by a thousand of products in pilot studies. Already, 65 studies are in Phase III and 21 studies are in combined Phase II/III. This suggests the near entry of some of those products. The development program (smaller sample sizes, single-arm trial designs, etc.) of such products may increase payers’ uncertainty about the product value at the time of launch. This uncertainty facing the payers and the important clinical benefits will contribute to the growing pressure put on payers during decision making. Coverage with evidence development with escrow agreements may become increasingly common in the pricing of ATMPs. The budget impact will possibly be considerable, threatening the sustainability of health insurance. The assessment of the expected clinical benefit, as well as the potential budget impact of several selected ATMPs, are the next steps of this project and have already been initiated.
